# Sex, Dose, and Equality

**DOI:** 10.1371/journal.pbio.0050340

**Published:** 2007-12-27

**Authors:** Brian Oliver

As a rule, genes and chromosomes come in pairs. Sex chromosomes are an exception to this rule. Males of many species have only one X chromosome, a male-specific Y chromosome, and a set of autosomes (AA). Individuals with two X chromosomes and a set of autosomes (XX;AA) are female. Sex chromosomes were first noticed for this distinct unpaired morphology and are now known to have substantially different gene content [1]. These unusual cases have attracted a great deal of attention over the years, not only because of the role they often play in sex determination, but also as windows into more basic features of genes and gene networks. One such feature is the relationship between gene function and dose. Sex chromosomes allow us to question the importance of having a pair of each gene. With current knowledge of gene regulation, one can make an argument that gene dose should not matter. In textbooks and manuscripts, one often finds figures showing the relationship between genes in a pathway or network, replete with elegant feed-back and feed-forward regulatory interactions, parallel pathways, etc. At the transcript level, it seems logical that any inherent 2-fold quantitative difference due to gene dose should be dwarfed, or even nullified, by the high-magnitude changes resulting from transcriptional regulation by proteins that are arrayed at enhancers or silencers. Basic textbook knowledge of genetics also suggests that dose is not very important. Having a single copy of most genes is not deleterious—there are few dominant alleles due to haploinsufficiency. These observations suggest that genes come in pairs to facilitate reproduction, and perhaps to provide a backup in case of spontaneous mutations occurring during the course of somatic development. It seems likely that the dose of most genes is unimportant because of robustness in gene networks, which buffers against noise and mutation [2].

Gene regulatory robustness probably makes cells and organisms insensitive to small differences in the dose of components, because such small deviations are also characteristic of more garden-variety noise. However, robustness has limits. Whereas the dose of individual genes does not appear to be very important, altering the doses of many genes is clearly detrimental. Chromosomal rearrangements are associated with many cancers. In some cases, these rearrangements break individual genes, but often there is a duplication or loss resulting in a change in copy number [3]. Monosomy (one rather than two chromosomes) or trisomy (three rather than two chromosomes) occur as a result of errors in meiosis and are highly deleterious during development of the resulting zygote [4]. With the exception of trisomy 21 and the sex chromosomes, altered chromosome dose is generally incompatible with human life. More recently, gene dose polymorphisms have been found to be relatively common in humans [5]. These copy number variations are the subject of a great deal of recent work, and may well be important factors in disease. In one of the first modern genomics papers, Drosophila researchers tiled the genome and showed that the viability of flies depends mostly on how much of the genome is present in one or three copies and not on the dose of individual genes [6]. Altering dose results in a mutant phenotype has an additive effect of small changes in the genome.

**Figure 1:**
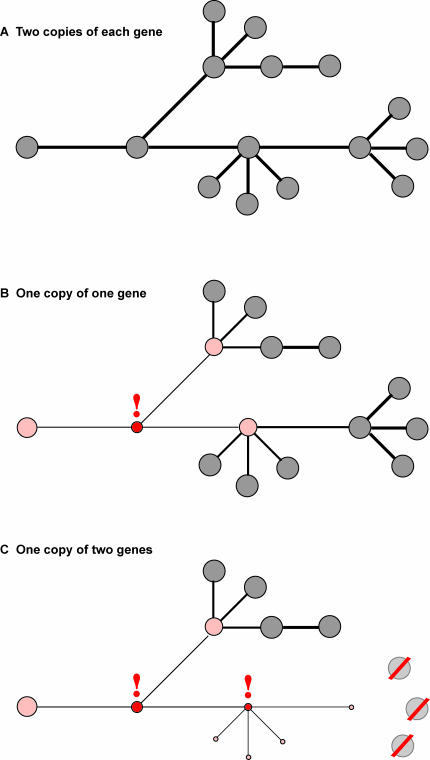
Network Cartoon (A) In this simple diagram, genes (circles) and regulatory connections (lines) are shown. (B) Reducing the dose of one gene (red exclamation point) perturbs the immediately connected genes (rose), but does not collapse the network. (C) Reducing the dose of two genes result in the loss of one part of the network (red slash).

To illustrate this point, take a small network (Figure 1A) where each node (a gene) has the same strength (dose). Quantitative disruptions at a single particular network node (a gene) might propagate through edges (gene activity) to adjacent nodes, but the structure of the network recovers with very little effect on the overall network structure (phenotype), as seen in Figure 1B. This is essentially the classic molecular biology model, where gene regulation restores proper network architecture in the face of small dose differences or significant environmental (e.g., heat shock) or biological (e.g., insulin) cues. Such networks are resistant to random suboptimization, but they are susceptible to disruption under concerted attack, for example, by geneticists trying to decipher a pathway. If the dose of enough genes is altered, as probably occurs in the case of something on the scale of monosomy, then the random disruption affects near neighbors by chance (Figure 1C). Gene expression levels are the result of the integration of multiple inputs that are quasi-redundant, but if enough of those inputs are weakened by a dose change, then there is a tipping point, the defect is propagated, and the network fails to recover. It is this network collapse, not the dose of individual genes, that results in a mutant phenotype.

Sex chromosomes are normally monosomic in the heterogametic sex (in XY systems where males have a single X, and in ZW systems where females have a single Z). This wild-type alteration is therefore a very powerful model for understanding how networks respond to gene dose—by showing us how the organism compensates for dose differences, how the network responds when compensation is absent, and how compensation evolves. The manuscript in this issue of *PLoS Biology* from Turner and colleagues [7] examines X chromosome dosage compensation in mammals.

**Figure 2:**
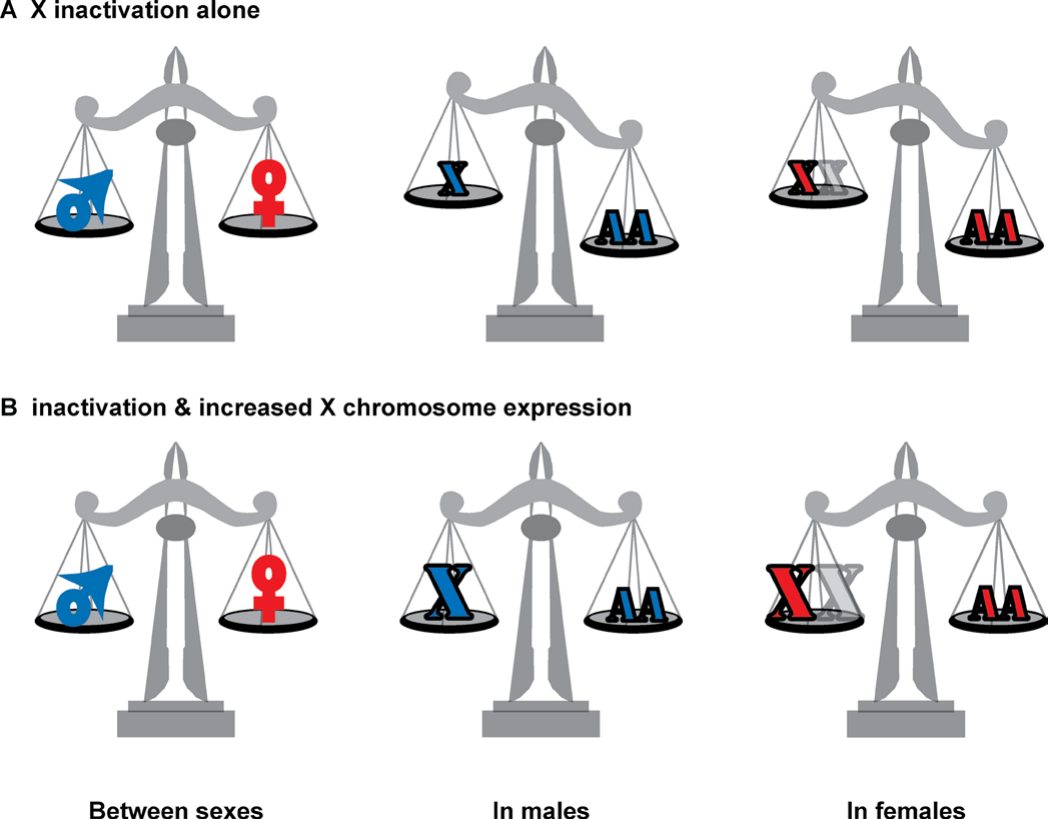
Balancing Expression (A) With X inactivation alone, female and male gene expression is equilibrated, but X and autosome (AA) expression in both males (blue) and females (red) is not. (B) X inactivation along with increased expression from the active X chromosome balances between the sexes and between X and autosome.

In mammalian females, one of the X chromosomes is condensed into largely inactive heterochromatin [8]. The idea is that both females and males are functionally monosomic for the X, and thus dose is compensated among the sexes (Figure 2A). This is an odd mechanism. In the science fiction story “Harrison Bergeron,” equality is mandated by law, such that particularly talented dancers are required to wear weights to disrupt their performances, and intellectually astute individuals have implants that periodically disrupt brain function [9]. Similarly, X inactivation achieves dosage compensation by giving females the same problems as males [10–12]. Unless the gene content of X chromosomes has become especially dosage tolerant (which would be quite interesting), then dosage compensation between the mammalian sexes would be expected to result in the same type of network collapse that might be expected due to monosomy for a major autosome. Simply put, X inactivation is a dosage-disrupting mechanism for females, not a dosage-compensation mechanism. What is bad for the gander is also bad for the goose.

It seems likely that X inactivation is not the whole dosage-compensation story in mammals. Work by Turner and colleagues [7] as well as by others [13,14] might necessitate the rewriting of some textbooks. These global expression-profiling studies show that X chromosomes are expressed in balance with autosomes in both females and males. This leads to the idea that the single X in males and that the single active X in females is over-expressed relative to a single autosome (X times two equals A plus A). This is an attractive idea, because it results in X/AA dosage compensation in both females and males (Figure 2B). X inactivation might be best seen as a response of females to the overexpression of the X chromosome, an overexpression that males require because they have just one X chromosome.

While this is a thematically pleasing idea that equilibrates expression between and within the sexes, the model represents a major paradigm shift, hinging on array analysis where overall X chromosome expression is compared to overall autosome expression. These data indicate that single active X chromosome expression rates (or more accurately, steady-state levels) are the same as those of two autosomes. While the datasets are quite large and in good agreement, the fold-changes are inherently modest. There is some autosomal dosage compensation in response to gene dose (buffering or network robustness), so X chromosome–specific dosage compensation results in much less than a 2-fold difference in expression [12,15]. There is also a counter example, where expression profiling indicates that sex chromosome dose in birds is poorly compensated [16]. At least some functions in bacterial chemotaxis are resistant to quantitative variation in component abundance [17]. Perhaps birds have particularly robust gene networks. Clearly, there is much room and demand for further work.

**Figure 3:**
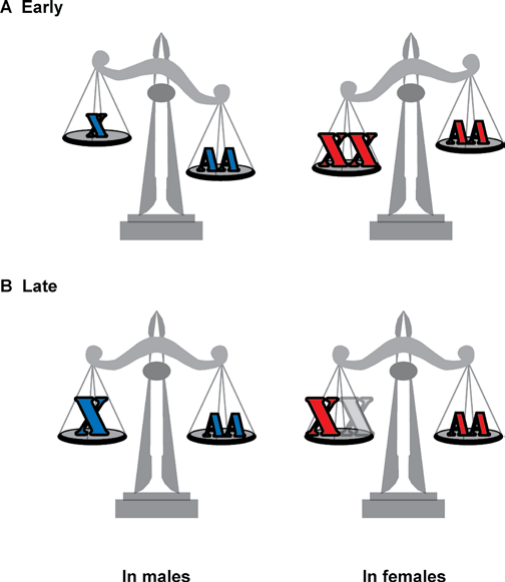
Dosage Compensation in Development, with Regulation in Both Sexes (A) Initially, X and AA expression are out of balance in both males and females. (B) The combination of increased X chromosome expression and X inactivation in females balances expression later in development.

Time-course analysis is a powerful gene-profiling tool, because having a vector in addition to a fold-change greatly increases confidence in small magnitude changes. Lin et al. [7] took advantage of the ability of male and female mouse embryonic stem cells to differentiate in culture to generate a time-course for dosage compensation. The shift in X chromosome expression relative to autosomes during the differentiation of male and female mouse embryonic stem cells is very convincing. This strengthens the case for increased expression of X chromosomes and shows how X chromosome dosage compensation is achieved as development proceeds. It is known that X chromosome inactivation occurs during early embryogenesis [8]. As would be expected under either the old X-inactivation–only or the new increased–X expression models of dosage compensation, there is higher X chromosome expression in female cells before X inactivation than in male cells. It is interesting that the expression of X chromosome genes is higher than autosomal expression in females, suggesting that X chromosomes are inherently hyperactive (Figure 3A). This in turn suggests that X inactivation is a mechanism for preventing functional tetrasomy in females. During the first week of development, X chromosome expression in females decreases toward balance with the autosomes, as a consequence of X inactivation (Figure 3B). The situation in males is something of a mirror image. Expression from the single X chromosome in male cells is lower than expression from the paired autosomes, but gradually increases until balance with the autosomes occurs (Figure 3). This suggests that X chromosome expression is ramping upward during early male development in order to come into balance with autosomal expression. Thus, at face value it appears that there are active regulatory mechanisms in both females and males.

While perhaps less likely, it is also possible that all the regulation occurs in females. It should be noted that the baseline expression of the single X in male embryonic stem cells is already expressed more than an autosome is before differentiation. This may be due to an inherent hyperexpression from the X chromosome. However, it may be more likely that this increased expression is the result of the buffering effects of networks [12]. Dose-dependent expression from cells with deletions on autosomes would provide a valuable reference for determining whether single X chromosomes are always overexpressed relative to single-copy regions on autosomes. Inherent hyperexpression of X chromosomes in both sexes (this certainly appears to be true for females) and regulated X inactivation in females can also result in true dosage compensation. Determining if X chromosome hyperexpression is gradually acquired in males or is a constant characteristic of X chromosomes will be important.

There is a large body of literature on the mechanism of X inactivation, which involves the expression of a noncoding RNA (*Xist*) from the X inactivation center, some type of *cis*-recognition of the chromosome to inactivate, chromatin modification, and condensation [8]. There is ongoing debate about how important entry sites for inactivation and spreading might be in this process. Entry sites and spreading occur in interphase cells, where chromosomes are arranged in characteristic territories within the spherical nucleus. Lin et al. [7] suggest that the gradual inactivation occurs in groups of genes by spreading into the X-chromosome territory. The idea that mosaic inactivation is dependent on the proximity to the *Xist* locus in three-dimensional chromosome-territory space is attractive, because spreading in three dimensions can explain interspersed regions on X chromosome that might fail to inactivate initially (imagine pouring sauce on spaghetti). The authors show that there is co-inactivation of neighboring genes on the X chromosome. However, there is a general correlation in gene expression of neighboring genes on all chromosomes, so it is not really clear if co-inactivation tell us much about X inactivation per se [18]. Understanding how inactivation at the transcript level is gradually acquired and how this relates to the previous observed changes in chromosome structure is important but will require the pairing of expression analysis with more systematic global analysis of *Xist* and modified histone localization by chromatin immunoprecipitation (ChIP) followed by array hybridization (ChIP-Chip) or deep sequencing (ChIP-Seq) [19,20]. Similarly, there is a clear need for high-throughput mapping techniques to provide a three-dimensional distance measurement between a given location on an X chromosome and another location on the X or any other chromosome [21].

Finally, the molecular mechanisms for increases in the expression of X chromosomes are wholly unknown. We just have questions. Do the active X chromosomes in females and males have a more active or less repressive chromatin structure? Is there something entirely novel that equilibrates steady-state expression of X chromosomes and autosomes? How much dosage compensation is needed on top of an inherent dose-response system that should function on autosomes? Studying the gradual acquisition of dosage compensation during early mammalian development may not provide all the answers, but it appears to be a good place to start.
